# DDKA-QKDN: Dynamic On-Demand Key Allocation Scheme for Quantum Internet of Things Secured by QKD Network

**DOI:** 10.3390/e24020149

**Published:** 2022-01-19

**Authors:** Liquan Chen, Qianye Chen, Mengnan Zhao, Jingqi Chen, Suhui Liu, Yongli Zhao

**Affiliations:** 1 School of Cyber Science and Engineering, Southeast University, Nanjing 210096, China; 220205039@seu.edu.cn (Q.C.); 220194603@seu.edu.cn (M.Z.); 220205041@seu.edu.cn (J.C.); 230219091@seu.edu.cn (S.L.); 2 Purple Mountain Laboratories for Network and Communication Security, Nanjing 211118, China; 3 School of Electronic Engineering, Beijing University of Posts and Telecommunications, Beijing 100876, China; yonglizhao@bupt.edu.cn

**Keywords:** QKD network, quantum Internet of Things, key allocation scheme, QKP

## Abstract

In the era of the interconnection of all things, the security of the Internet of Things (IoT) has become a new challenge. The theoretical basis of unconditional security can be guaranteed by using quantum keys, which can form a QKD network-based security protection system of quantum Internet of Things (Q-IoT). However, due to the low generation rate of the quantum keys, the lack of a reasonable key allocation scheme can reduce the overall service quality. Therefore, this paper proposes a dynamic on-demand key allocation scheme, named DDKA-QKDN, to better meet the requirements of lightweight in the application scenario of Q-IoT and make efficient use of quantum key resources. Taking the two processes of the quantum key pool (QKP) key allocation and the QKP key supplement into account, the scheme dynamically allocates quantum keys and supplements the QKP on demand, which quantitatively weighs the quantum key quantity and security requirements of key requests in proportion. The simulation results show that the system efficiency and the ability of QKP to provide key request services are significantly improved by this scheme.

## 1. Introduction

The Internet of Things (IoT), composed of numerous heterogeneous devices, has realized a convenient and efficient communication between things located in physically unconnected places [1,2]. Due to the peculiar requirements of IoT, guaranteeing the credibility and data security of the IoT still faces several challenges [3,4]. In the current IoT system, a relatively simple method of data encryption, commonly known as lightweight cryptography, is adopted to guarantee the security of data transmission. However, this method has the risk of being cracked by quantum computers [5], which will lead to a series of challenges, especially those related to the privacy and security of the IoT.

Quantum key distribution (QKD) has the potential to provide long-term security for communication. Due to the basic law of quantum physics [6], for example, No-Cloning Theorem, uncertainty principle, etc. Distributing keys by the QKD mechanism can effectively realize the security of data transmission [7,8]. A quantum key distribution network (QKDN) formed by multiple point-to-point QKD systems, can be used to provide a remote quantum key service for multiple users.

Under the traditional IoT architecture, combined with the characteristics of QKDN, the layered system of quantum Internet of things (Q-IoT) is realized. On the basis of the three-layer structure of the traditional IoT: application layer, network layer and perception layer, Q-IoT adds a quantum security layer [9]. The application layer processes the data securely based on the quantum keys. The network layer provides service support for quantum key distribution of quantum backbone network, metropolitan area network and access network. The perception layer realizes the quantum key distribution between the edge gateway and the IoT terminal. The quantum security layer realizes the centralized management and efficient scheduling of the quantum keys for the entire IoT.

In the application scenario of Q-IoT, due to the low generation rate of quantum keys, in order to obtain the quantum key more efficiently, the QKD network uses the QKPs at the edge gateway to store the quantum keys. Therefore, in face of a large number of IoT requests, the quantum key is a scarce resource. How to allocate quantum key resources to satisfy the efficiency and lightweight requirements of Q-IoT applications is an urgent problem to be solved.

### 1.1. Related Works

The QKDN involves the efficiency problem of quantum key allocation. The QKDN involves the efficiency problem of quantum key allocation. Many researchers have reconstructed the scheme based on the research of classical key allocation schemes and the application scenarios of QKD on this problem. For the elaboration of the problem of quantum key allocation, we analyzed the related work developed in recent years.

Niu et al. [10] proposed a scheme of key size-driven wavelength assignment (KSD-WA), which reclaims the wavelength segments to transmit the quantum signals, thus, the wavelength of the quantum channels may require reconfiguration at different time slots if required. Taking the QKP technique in the QKD network into account, KSD-WA optimizes it with a heuristic algorithm and designs a deep reinforcement learning-based algorithm to optimize the fragment selection. The choice of wavelength maximizes the security key rate in dynamic scenarios.

Wang et al. [11] constructed intra-domain key pools for nodes within arbitrary domains, as well as inter-domain key pools for nodes at domain boundaries and proposed a capacity adaptive supplementary scheme based on the balance between key resources and routing, which reduces the service congestion rate and improves the utilization of key resources.

In the application scenario of the IoT, a RAKP scheme was proposed by Meng et al. [12] in which the quantum key generation rate of a pair of QKD devices mainly depends on the receiving rate of QKD receivers. Considering that the QKD receivers of each optical line terminal (OLT) generate quantum keys for the QKPs, RAKP is proposed to make rational use of OLT quantum receivers to dynamically adjust the quantum key generation rate according to the utilization rate of quantum keys.

Cao et al. [13] proposed the KoD scheme using the RWKA algorithm to allocate the routing and wavelength by the data channel and used the First-Fit algorithm to allocate the keys by the control channel and the data channel. The adaptive key assignment strategy considers two cases, i.e., key-updating based on time complexity and data complexity. Simulation results show that KoD is beneficial to achieve the balance of security requirements and key resource usage.

A dynamic key configuration (DSKP) scheme was proposed to assign keys stored in the QKP of OLT and optical network unit (ONU) to users by Wang et al. [14]. DSKP scheme effectively generates and assigns keys from users’ demands. When the number of keys in QKP is less than the threshold, the secret-key-generation process is triggered to release the “Supplementary Request” to obtain the required key quantity in QKP. The secret-key-consumption process clusters secret-key-provisioning requests with the same destination nodes.

Cao et al. [15] proposed a new multi-tenant QKD network architecture and key rate sharing scheme based on Software Defined Network (SDN) and QKP technology, and then designed a heuristic algorithm to realize efficient multi-tenant key assignment on the QKD network. Based on each QKD tenant request, if the secret-key-rate demands can be satisfied, the algorithm selects the required secret-key-rate slots to form the corresponding QKD node pairs with the First Fit algorithm.

In 2019, Cao et al. [16] proposed an on-demand key resource allocation strategy based on SDN for multi-tenant configuration on the metro QKD network. The First-Fit algorithm is used to select the required key resource from the corresponding key server of QKD. The key resources are assigned according to the tenancy request.

A QaaS oriented SDN framework was proposed by Cao et al. [17]. Based on SDN technology, more efficient QKD network management is obtained when multiple users apply for quantum key services. The protocol extension, intercommunication workflow, and routing and secret-key-rate assignment strategy are presented for QaaS implementation over the QKD network.

Zuo et al. [18] introduce the reinforcement learning model and designs a heuristic quantum key resource distribution method based on best fitting so that each security service can choose a resource allocation method with higher long-term return according to the current resource usage of the network, so as to improve the stability of the QKP and the utilization of quantum key resources.

A comparison of the existing works with our scheme is shown in Table 1. In this table, ‘×’ means this issue has not been discussed in the paper while ‘√’ means this issue has been discussed in the paper. In this comparison, we list the advantages and disadvantages of each scheme.

Based on current researches, it can be derived that the QKD network still faces the following difficult problems in the application scenario of IoT:

1. From the perspective of IoT terminal devices, specific scenarios of the IoT have their own unique security requirements. Current research does not make a reasonable and efficient allocation of quantum key resources considering the difference of security requirements among IoT applications, which will reduce the overall service quality.
2. There are numerous and heterogeneous terminal devices accessed in the IoT. Due to the storage capacity limitations of many terminal devices of the IoT and the high cost of QKD device deployment, it is unable to store quantum keys on IoT terminal devices. Therefore, the efficiency of the IoT system to allocate quantum key resources is low especially facing a large number of quantum key requests.
3. From the perspective of QKD network, due to the low generation rate of the quantum keys, the QKD network adopts the accumulation of the quantum key resources to satisfy numerous IoT key requests by storing quantum keys in QKPs. Dealing with the security issue of the QKP storing quantum keys, the current research does not take into account the efficiency of the QKP responding when confronting a large number of IoT key requests.

### 1.2. Contributions and Organizations

In response to the above challenges faced by the QKD network in the application scenario of IoT, this paper mainly involves the following contributions:

1. In the QKD-based Q-IoT security protection system, quantum key distribution is achieved with QKDN and trusted relay technology, and the key storage management of QKP is realized with SDN technology. Moreover, based on the requirements of the IoT application for quantum key quantity and security, a reasonable allocation method of quantum key resources is proposed in this paper.
2. A dynamic on-demand key allocation scheme is designed to allocate quantum key resources. Specifically, quantum key resources are distributed more efficiently by considering the arrival time of the key requests, the key quantity and security requirements. The number of requests arriving at the same time can be large, resulting in a long queuing delay, which involves the prioritization of queues. Therefore, the scheme designs the response weight of requests, which quantitatively determines the response order based on the quantum key quantity and security requirements in proportion. The scheme is designed to better meet the requirements for efficiency and lightweight of the Q-IoT in the application scenarios.
3. In our scheme, the quantum keys of the QKP are dynamically supplemented. In the key resource supplement stage, the quantum keys are replenished in sequence based on the response weight of the key supplement request. Additionally, in consideration of the time slice resources, the remaining key amount of the QKP, and the key supplement request, a reasonable key supplement threshold is set to maximize the use of network resources and improve the ability of the QKP to provide quantum key services.

This paper is organized as follows: Section 1 introduces the background of Q-IoT and the related research of key resource allocation in the field of quantum communication. A quantum key distribution framework in the IoT scenario is presented in Section 2. In Section 3, a dynamic on-demand key allocation scheme for Q-IoT is demonstrated. The simulation experiments of the dynamic on-demand key allocation scheme and the analysis of improvement effect are detailed in Section 4. Section 5 analyzes the performance and summarizes the paper.

## 2. Quantum Key Distribution Framework for IoT

### 2.1. QKD Mechanism

**Figure 1:**
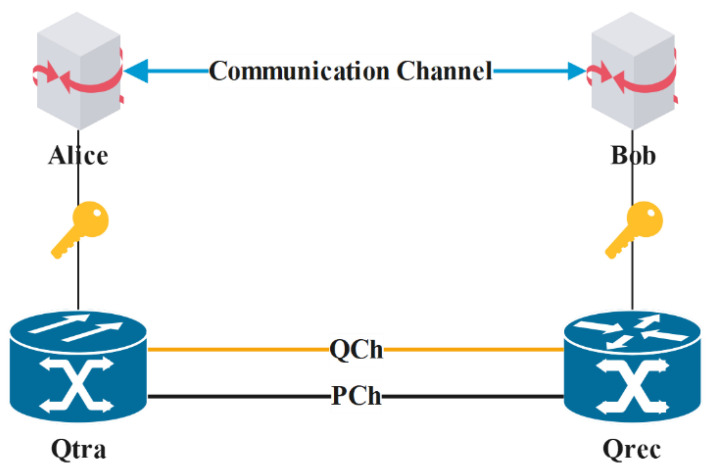
Schematic diagram of QKD.

Here, the QKD mechanism used in this paper is the BB84 protocol first proposed by Bennett et al. [19] in 1984. Different from the classical cryptosystem, the BB84 quantum key distribution protocol is based on the principles of quantum mechanics to ensure the security of point-to-point key distribution. The schematic diagram of the process is shown in Figure 1. Some abbreviations and their descriptions used in the following sections are listed in Table 2.

The schematic diagram of QKD shown in Figure 1 involves the exchange of quantum qubits between the quantum transmitter (Qtra) and the quantum receiver (Qrec) through the quantum channel (QCh), then exchanges the measurement base through the public channel (PCh). Next, the key is screened. Finally, the errors are corrected to determine whether there is an attacker and determine the final session key.

### 2.2. Quantum Key Storage

Because the storage resources of IoT terminal devices are limited and the generation efficiency of the quantum keys is low, the accumulation of quantum key resources will be used to satisfy the quantum key usage. A QKP is placed at the edge gateway to store the quantum keys used for communication between IoT devices. When the IoT terminal device needs encrypted communication, it initiates a key request to the edge gateway. Then, the edge gateway extracts the quantum key from the QKP and transmits it to the IoT terminal device.

The key storage device is abstractly virtualized as a QKP, which exists between any two QKD nodes. The keys are managed in pairs between the two nodes. According to the source node and the destination node of the communication, the QKP can be divided into multiple virtual spaces to become virtual key pools (VKP), which are specifically identified by indexes. The keys are placed in the VKP corresponding to the index number according to the source node and the destination node of the communication requests, which meets the security requirements of the communication parties for one-to-one key resource distribution. In this way, the QKPs at the edge gateway nodes can be divided into two virtual key pools according to different communication scenarios, one is the internal key pool and the other is the cross key pool. The internal key pool that refers to the key generation in the QKP involves only one OLT while the cross key pool that refers to the key generation in the QKP involves two OLT optical line terminals [12].

**Figure 2:**
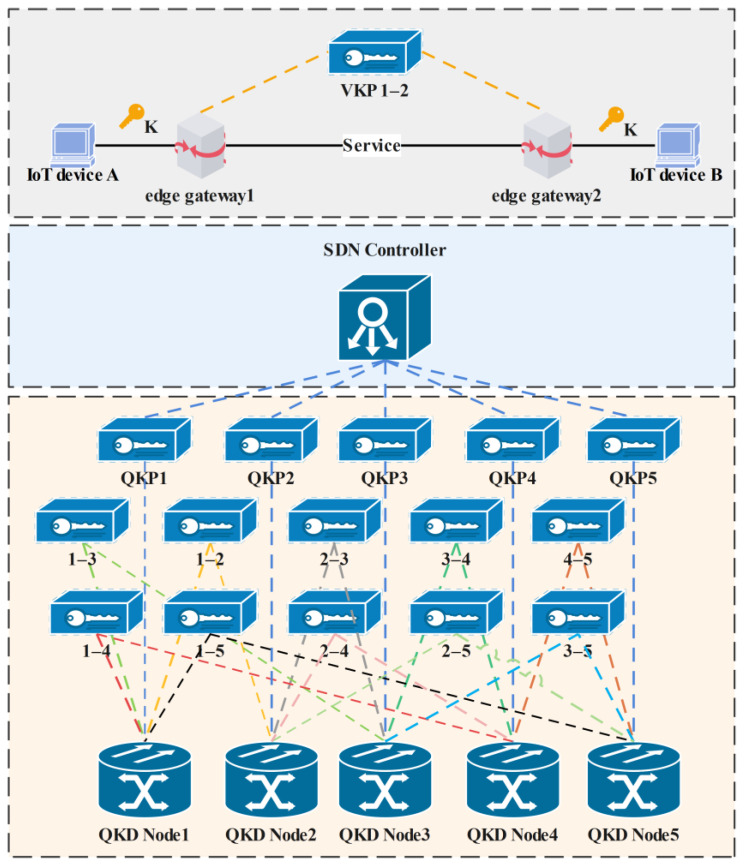
Schematic diagram of QKP architecture based on SDN.

The construction of QKP and VKP adopts software-defined network (SDN) technology [20] to flexibly schedule key resources according to communication requests. The overall structure is shown in Figure 2.

### 2.3. Quantum Key Distribution Scenario

The quantum transceivers cannot be placed in IoT terminals because the QKD technology is limited at this stage and the IoT terminals are numerous. Moreover, from the perspective of deployment cost, computing resources, security and credibility, quantum transceivers can only be placed in metropolitan nodes and access network nodes, etc.

Therefore, to realize the quantum key distribution from the access network to the IoT terminal devices, a quantum receiver Qrec and a trusted quantum relay device are arranged at the OLT, which can be shared by multiple quantum transmitters Qtra [21] for key generation. The quantum transmitter Qtra is arranged at the ONU to realize the point to multi-point quantum key distribution from the access network to the edge gateway. Long-distance communication needs to overcome the influence of transmission medium on signal loss. So, to solve the problem of signal loss of quantum signal in the quantum channel during long-distance transmission, the key trusted relay technology is used to provide end-to-end quantum keys with trusted repeaters [22,23]. The relay key generated by the point-to-point QKD link can be XOR-encrypted to relay it to the target node. The final session key is not transmitted directly. The node only transmits the quantum key processed by XOR encryption technology. The receiver decrypts it with the shared symmetric key to obtain the quantum key finally used for the session, which can achieve high security end-to-end key distribution.

**Figure 3:**
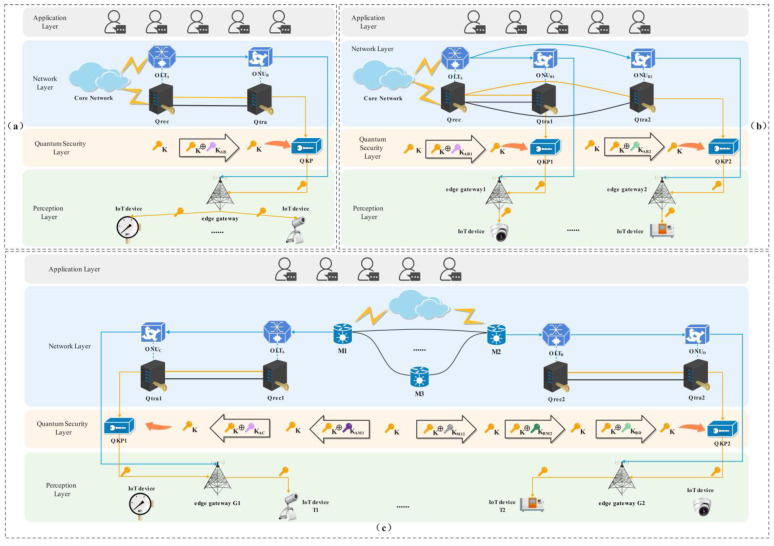
(**a**) The communication between devices under the same edge gateway; (**b**) The communication between devices under two edge gateways of the same OLT; (**c**) The communication between devices under two edge gateways of different OLTs.

The following three different Q-IoT communication scenarios are considered respectively: (a) the communication between IoT devices under the same edge gateway; (b) the communication between IoT devices under two edge gateways of the same OLT; (c) the communication between IoT devices under two edge gateways of different OLTs. A schematic diagram of a specific key distribution scenario is shown in Figure 3.

Take the communication between devices under two edge gateways of different OLTs as an example. As shown in Figure 3c, the device T1 under the edge gateway G1 of OLT_A_ and the device T2 under the edge gateway G2 of OLT_B_ communicate: when they are under different metropolitan area nodes, the relay process between metropolitan area nodes is required. The key K_M12_ is shared between metropolitan area node M1 and metropolitan area node M2. When the M1 receives the quantum key K, it performs the XOR encryption K ⨁ K_M12_ on the quantum key K. The M2 uses the key K_M12_ shared with M1 to decrypt to obtain the quantum key K. At this time, both M1 and M2 obtain the quantum key K.

Next, M1 and the OLT_A_ share the key K_AM1_. The M1 performs XOR encryption K ⨁ K_AM1_ on the quantum key K. The OLT_A_ uses the key K_AM1_ shared with M1 to decrypt the quantum key K. The OLT_A_ and the ONU_C_ share key K_AC_. The OLT_A_ then performs XOR encryption K ⨁ K_AC_ on the quantum key K. The ONU_C_ decrypts the quantum key K with the key K_AC_ shared with the OLT_A_. The quantum key K is stored in the QKP corresponding to the edge gateway G1. The quantum key K in the QKP corresponding to the edge gateway G2 is transmitted in the same way.

Based on the principle of OTP, the quantum key distribution from edge gateways to IoT terminal devices is realized by physical layer key generation technology [24]. The IoT devices and the edge gateways extract channel features on the wireless channel of communication to generate keys. Then, the quantum keys are encrypted by the encryption key and sent to the IoT terminals through the wireless channel. Finally, the IoT mobile terminals obtain the quantum key by decryption.

It should be noted that all relay nodes are required to be trustworthy. At the same time, all quantum keys used for the final session are not transmitted directly. They are transmitted after being processed by XOR encryption technology, which increases the security of key transmission.

## 3. The Proposed DDKA-QKDN Scheme

### 3.1. Scheme Characteristics

QKD network will face many problems in the application scenario of the IoT. Firstly, when encountering a large number of quantum key requests, due to the limitation of storage and computing capabilities of many IoT terminal devices, it puts forward high requirements on how to efficiently allocate quantum key resources. In addition, the storage security problem of quantum keys in the QKPs needs to be solved. Furthermore, because of the limitation of the storage capacity of IoT devices, a QKP is used at the edge gateway to store quantum keys. When dealing with the distribution of quantum key resources, the supplement of quantum keys of the QKPs needs to be considered.

Therefore, according to the requirements of IoT applications, it is of great significance to allocate quantum key resources efficiently to reduce the response delay of quantum key requests, improve IoT system efficiency and even reduce the time cost of receiving quantum keys for IoT terminal devices.

Considering a general application scenario in the Q-IoT, quantum keys with different bits are selected for encrypted communication according to the different security requirements of IoT applications. After the IoT application arrives, the IoT terminal device node sends a quantum key request to the edge gateway and queues up for the QKP to respond to the key request, and then obtains the quantum keys from the QKPs. When the remaining key amount of the QKP does not meet the required number of keys, the QKP needs to send a key supplement request for the key replenishment. Because the number of requests arriving at the same time could be potentially huge, there will be a large queuing delay. Therefore, the efficiency problem needs to be solved due to the lightweight characteristics of the IoT.

The proposed dynamic on-demand key allocation scheme, DDKA-QKDN, considers the key allocation and the key supplement of the QKP simultaneously. In the key allocation phase of the QKPs, the remaining keys in the QKPs are allocated on demand based on the arrival time of the key requests, the quantum key quantity requirements, the security requirements of the quantum keys, and the remaining keys quantity in the QKPs. In the key supplement phase of the QKPs, the keys are supplemented sequentially based on the response weight of the key supplement requests. At the same time, the keys in the QKPs are dynamically supplemented in the idle time slot. The goal of the DDKA-QKDN scheme is to achieve a balance between quantum key resources and the security requirements of IoT terminal applications, further improving the IoT system efficiency as a whole. Some mathematical symbols and their descriptions used in the following sections are listed in Table 3.

### 3.2. Key Request Security Classification

In response to the requests of IoT applications, combined with the characteristics of lightweight data processing of IoT itself, lightweight data acquisition and message management are adopted to classify the security level of the messages. According to different security levels, the quantum key security requirements for information encryption of IoT applications are quantitatively determined.

According to the security requirements of IoT applications, the security level *Sec* is classified. First, select whether quantum key communication is required. Based on the lightweight requirements of the IoT and system efficiency, the use of the quantum key is only for the case when the security requirements of message applications are high. For the case of a low-security level (*Sec* = 0), the message is directly transmitted in plaintext. For the case of a high-security level (*Sec* > 0), the quantum keys of different lengths can be selected for secure communication according to the security level *Sec*. The lengths of quantum keys are 128 bits, 256 bits, 512 bits, 1024 bits, and 2048 bits.

### 3.3. Quantum Key Allocation on Demand

**Figure 4:**
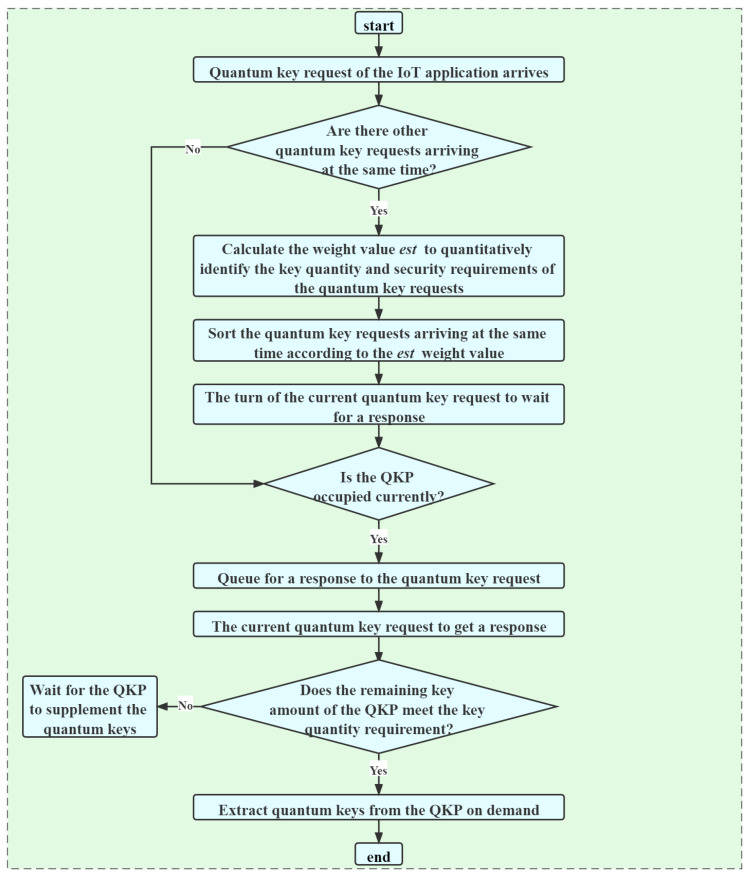
Flow chart of responding to key requests.

The on-demand key allocation process of the QKPs is that after the application key requests arrive, multiple IoT terminal devices send multiple key requests to the edge gateway. The key requests need to include quantum key quantity requirements, quantum key security requirements, and other information. After receiving multiple key requests, the edge gateways extract the quantum keys from the corresponding QKPs at the edge gateway. The specific process is shown in Figure 4.

There are many IoT devices, the QKP may process a large number of IoT key requests, so the DDKA-QKDN scheme designs a queuing response sequence as follows:

1. The highest priority is the arrival time. The QKP responds to the key requests in order according to the arrival time of the key requests.
2. The second highest priority is the response weight value of the key requests, which is quantified by a certain weight $est\left(K_{i}\right)$ to identify the key quantity requirements and key security requirements of the key requests, that is, the key requests in the case of the same arrival time, weigh the key quantity requirements and key security requirements to determine the key allocation order. The calculation equation of the response weight value $est\left(K_{i}\right)$ is as follows:
  (1) $$est\left(K_{i}\right)=\left(1-\omega \right)lnK_{qua}+\omega ln\left(10-K_{sec}\right).$$
  where $K_{qua}$ represents the quantum key quantity requirement, $K_{sec}$ represents the quantum key security requirement, ω∈[0,1], which represents the trade-off degree of the quantity requirements and the security requirements of the key services. By adjusting the size of ω, the different requirements of the key quantity and the security of the key requests are met. The response weight value $est\left(K_{i}\right)$ of the key request is arranged in ascending order. In consideration of the system efficiency, the smaller the key requirement $K_{qua}$, the higher the priority of the key request. From the perspective of system security, the higher the key security requirement $K_{sec}$, the higher the priority of the key request.

Based on the queuing response sequence requirements of the above key requests, two different application scenarios are considered according to the remaining key amount of the QKP:

1. If the quantum remaining key quantity of the QKP is sufficient to meet the key quantity requirements of the arriving key requests, the keys are allocated according to the queuing response sequence of the key requests.
2. Otherwise, the edge gateway sends a quantum key supplement request of the QKP. After waiting for the QKP to supplement the keys and when the remaining key quantity of the QKP can satisfy the key quantity requirement of the key requests, the key allocation of the key request is performed.

### 3.4. Quantum Key Supplement

**Figure 5:**
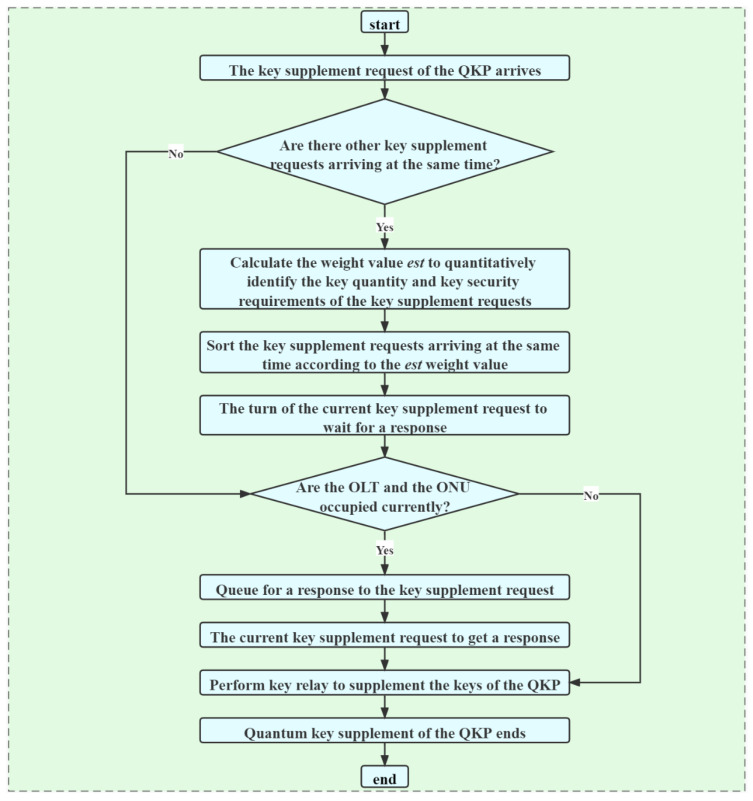
Flow of responding to key supplement requests.

The key supplement process of the QKP consists of two parts. One part is that when the remaining key quantity of multiple QKPs is not enough to provide key services, the QKP sends a key supplement request, which needs to include quantum key quantity requirements, quantum key security requirements, and other information. According to the key supplement request, keys are generated between the OLTs and the ONUs to supplement the QKP. The specific process is shown in Figure 5.

The key generated between the OLTs and the ONUs can only be relayed one-to-one at the same time. Therefore, when responding to multiple QKP key supplement requests, the DDKA-QKDN scheme designs the queuing response sequence of key supplement requests, which is detailed as follows:

1. If the quantum remaining key quantity of the QKP is sufficient to meet the key quantity requirements of the arriving key requests, the keys are allocated according to the queuing response sequence of the key requests.
2. The second priority is the response weight value of the key supplement request, which is the same as the response weight value of the key request. When the arrival time of the key supplement request is the same, it is used to determine the order of key supplements by balancing the requirements of key quantity and key security. The calculation equation of the response weight value $est\left(K_{i}\right)$ is as follows:
  (2) $$est\left(K_{i}\right)=\left(1-\omega \right)lnK_{qua}+\omega ln\left(10-K_{sec}\right)$$

Here ω∈[0,1] and it represents the trade-off degree of the system’s key supplement requirements for the quantity requirements and the security requirements. By adjusting the size of ω, the different requirements of the key quantity and the security of the key supplement requests are met.

Another part of the key supplement process of the QKP is to dynamically supplement the keys in the QKP in the idle time slot. Due to the dynamic and suddenness of key services, the generation and consumption of key resources are often unbalanced. If the quantum key resources in the QKP are consumed too fast, it may reduce the success probability of subsequent key services. Conversely, if the quantum key resources are consumed slowly, the quantum key resources in the QKP may accumulate gradually. When the quantum key resources are stored in the QKP for a long time, it will increase the risk of quantum key disclosure and reduce the security of quantum keys.

In order to solve the above two problems of key storage, the thresholds of QKP are set up. When the remaining key amount is too small and is lower than the set low threshold, to prevent the remaining key quantity of the QKP from being unable to meet the key quantity requirement of the subsequent key requests, the keys are supplemented in time. At the same time, when the number of remaining keys is too large and higher than the set high threshold, to prevent too many quantum keys from being stored for too long, the security of quantum keys will be reduced, and the supplement of quantum keys will be stopped. When in the range of the two thresholds, the quantum keys of the QKP are dynamically supplemented according to the key quantity requirements and security requirements.

### 3.5. Scheme Detail

The main idea of the DDKA-QKDN scheme is to dynamically allocate quantum key resources according to the application requirements. The following is an analysis of the scheme features from two perspectives, efficiency and security, specifically explaining how each feature affects the way of key resource allocation.

1. System efficiency: Because there are a large amount of IoT devices, the IoT key requests arriving at the same time will cause the rapid consumption of network resources and the risk of congestion. To minimize the waiting delay after the IoT key request arrives, the two main factors that affect the system efficiency, the key quantity requirement $K_{qua}$ and QKP remaining key amount $K_{sur}$, should be considered primarily. From the perspective of application requirements, a higher priority is given to the key request with a smaller $K_{qua}$ value. Because it takes less processing time, the waiting time can be reduced for subsequent key requests. When it comes to key resource storage, the scheme considers that when the remaining key amount $K_{sur}$ of the QKP is lower than the low threshold $K_{threshold\_low}$ of the QKP, the keys are supplemented in time. This can reduce the time for key requests to wait for the keys to be supplemented and increase the number of key requests that can be responded to in time.
2. Security: Because the security requirements of IoT key requests are different, and due to the limitations of the computing and storage resources of the IoT, the quantum keys are stored in the QKPs in advance, the scheme considers the security of the system in terms of application requirements and key resource storage. In terms of application requirements, the scheme considers the security requirement $K_{sec}$ of key requests as a dimension to be considered for the queuing response sequence of the key requests and the key supplement requests. The key requests with higher $K_{sec}$ values are given higher priority. In terms of key resource storage, when the QKP performs the key supplement, if the high threshold $K_{threshold\_high}$ of the QKP is exceeded, the key supplement is stopped. This can reduce the risk of key leakage in the QKP.

The detailed steps of the scheme are shown in Table 4 below.

After the IoT application key requests $K_{Request}$ arrive, multiple IoT devices send multiple key requests $K_{Request}$ to the edge gateway G1. The key request $K_{Request}$ must contain the quantum key quantity requirement $K_{qua}$, quantum key security requirements $K_{sec}$, ID of both sides of the session, namely the session application terminal T1, the session target terminal T2 and the edge gateway G1 corresponding to T1 and the edge gateway G2 corresponding to T2, etc. That is, $K_{Request}=\left(K_{qua},K_{sec},T1,T2,G1,G2\right)$.

The arrival of the key requests $K_{Request}$ obeys the Poisson distribution, which can simulate and describe the network data traffic. The probability that there are n key requests $K_{Request}$ arriving in the $t_{slot}$ time slot is:

(3) $$P=\left(N\left(t_{slot}\right)=n\right)=\frac{{\left(\lambda t_{slot}\right)}^{n}}{n!}e^{-\lambda t_{slot}}.$$

In Equation (3), λ represents the arrival frequency of the key requests $K_{Request}$, $t_{slot}$ can be regarded as a fixed value within a certain period of time. According to the characteristics of Poisson distribution, the average key stream load $L_{req}$ that can be carried in the $t_{slot}$ time slot is:

(4) $$L_{req}=E\left(L\left(t_{slot}\right)\right)=\lambda \times t_{slot}$$

When the remaining key quantity $K_{sur}$ of the QKP cannot satisfy the requirement of the key requirement $K_{qua}$ of the current key request $K_{Request}$, it is necessary to send a key supplement request $K_{Supplement}$ to the OLT. The key supplement request $K_{Supplement}$ shall include information on the QKP and the quantum key requirement $K_{qua}$ and quantum key security requirement $K_{sec}$ of current key request $K_{Request}$, ID of both sides of the session, namely the session application terminal T1, the session target terminal T2 and the edge gateway G1 corresponding to T1 and the edge gateway G2 corresponding to T2, etc. That is, $K_{Supplement}=\left(QKP,K_{Request}(K_{qua},K_{sec},\;T1,T2,\;G1,\;G2\right))$.

For incoming key request $K_{Request}$ and key supplement request $K_{Supplement}$, the highest priority of the response order is the arrival time. In the case of the same arrival time, the key quantity requirements $K_{qua}$ and security requirements $K_{sec}$ for key services are considered to determine the response order for the requests. A certain weight $est\left(K_{i}\right)$ is used to quantitatively identify the key quantity requirement $K_{qua}$ and the key security requirements $K_{sec}$:

(5) $$est\left(K_{i}\right)=\left(1-\omega \right)lnK_{qua}+\omega ln\left(10-K_{sec}\right)$$

In Equation (5), ω∈[0,1]. The scheme quantitatively considers the quantum key quantity requirements and quantum key security requirements of key requests according to the proportion and calculates the $est\left(K_{i}\right)\;$ value corresponding to each request. Based on this criterion, the queuing response order of key requests with the same arrival time is determined. The weights $est\left(K_{i}\right)$ are arranged in ascending order, the smaller the key requirement $K_{qua}$, the higher the security requirement of the key service $K_{sec}$, the higher the priority of the request. The ranking strategy aims to better meet the requirements of efficiency and lightweight in the application scenario of Q-IoT.

Furthermore, when the QKP, the OLT and the ONU are in the idle time slot, the dynamic key supplement is carried out for the QKP. Accordingly, two thresholds of the QKP, low threshold $K_{threshold\_low}$ and high threshold $K_{threshold\_high}$ are set. When the remaining key quantity $K_{sur}$ of the QKP is less than the low threshold $K_{threshold\_low}$, the QKP is supplemented to the QKP remaining key quantity $K_{sur}$ = the high threshold $K_{threshold\_high}$. The remaining key quantity $K_{sur}$ of the QKP is:

(6) $$K_{sur}\left(nt_{slot}\right)=K_{sur}\left(\left(n-1\right)t_{slot}\right)+nt_{slot}V_{gen},\;\;\;\;\;\;\;\;\;\;K_{sur}\left(\left(n-1\right)t_{slot}\right)<K_{threshold_{low}};$$

(7) $$K_{sur}\left(nt_{slot}\right)=K_{sur}\left(\left(n-1\right)t_{slot}\right),\;\;\;\;\;\;\;\;\;\;\;\;\;\;\;\;\;\;\;\;\;\;\;\;0<K_{sur}\left(nt_{slot}\right)\leq K_{threshold_{high}}.$$

In the idle time slot, as shown in Equation (6), the remaining key amount of the QKP for each time slot is judged. When the remaining key amount $K_{sur}$ is less than the set low threshold $K_{threshold\_low}$, the remaining key amount $K_{sur}$ in the QKP is increased. At the same time, as shown in Equation (7), it is judged whether the remaining key quantity $K_{sur}$ of the QKP in the time slot is within an interval less than the set high threshold $K_{threshold\_high}$. If this interval is exceeded, the key supplement will be stopped, and the remaining key amount $K_{sur}$ in the QKP remains unchanged.

Based on the above scheme architecture, the dynamic on-demand key allocation scheme considers three parts of the time delay: the queuing delay $t_{wait}$ of key requests waiting to obtain the key, the queuing delay $T_{wait}$ of QKP waiting for key supplement and the key transmission delay $T_{tra}$. The delay calculation of key requests is divided into two circumstances according to the actual key resources. One is that the number of remaining keys in the QKP meets the key quantity requirement of the key request and the QKP does not need to be supplemented, as shown in Equation (8); The other is that the amount of remaining keys in the QKP does not meet the key quantity requirement of the key requests and the QKP needs to be supplemented, as shown in Equation (9).

(8) $$T_{sum}=t_{wait}+T_{tra}=\{\begin{array}{c} K_{qua}\times t_{tra},\;\;\;\;\;\;\;\;\;\;\;\;t_{get}\leq t_{arr};\\ \left(t_{get}-t_{arr}\right)+K_{qua}\times t_{tra},\;\;\;\;\;\;t_{get}>t_{arr}. \end{array}$$

where $t_{get}$ represents the time when the last key request $K_{Request}\left(i-1\right)$ obtained the keys from the QKP, $t_{arr}$ represents the arrival time of the current key request $K_{Request}\left(i\right)$, and $t_{tra}$ represents the link transmission rate. According to the time slice resource occupancy, if $t_{get}>t_{arr}$, it means that the time slot of the QKP is occupied by the previous key request $K_{Request}\left(i-1\right)$, and the current key request $K_{Request}\left(i\right)$ needs to queue up to wait for the time slot to be released. If $t_{get}\leq t_{arr}$, it means that the current key request $K_{Request}\left(i\right)$ gets the response directly, and the quantum keys can be extracted from the QKP without queuing.

(9) $$T_{sum}=t_{wait}+T_{wait}+T_{tra}=\{\begin{array}{c} \left(t_{get}-t_{arr}\right)+(T_{get}-T_{arr})+\frac{K_{qua}-K_{sur}}{V_{gen}}+K_{qua}\times t_{tra}\\ ,t_{get}>t_{arr}\wedge T_{get}>T_{arr};\\ \left(t_{get}-t_{arr}\right)+\frac{K_{qua}-K_{sur}}{V_{gen}}+K_{qua}\times t_{tra}\\ ,t_{get}>t_{arr}\wedge T_{get}\leq T_{arr};\\ (T_{get}-T_{arr})+\frac{K_{qua}-K_{sur}}{V_{gen}}+K_{qua}\times t_{tra}\\ ,t_{get}\leq t_{arr}\wedge T_{get}>T_{arr};\\ \frac{K_{qua}-K_{sur}}{V_{gen}}+K_{qua}\times t_{tra}\\ ,t_{get}\leq t_{arr}\wedge T_{get}\leq T_{arr}. \end{array}$$

where $T_{get}$ represents the time when the last QKP key supplement request $K_{Supplement}\left(i-1\right)$ obtained the key, $T_{arr}$ represents the arrival time of the current QKP key supplement request $K_{Supplement}\left(i\right)$, and $V_{gen}$ represents the generation rate of the keys between the OLT and the ONU. According to the time slice resource occupancy, if $T_{get}>T_{arr}$, it means that the time slot of the OLT relay is occupied by the previous key supplement request $K_{Supplement}\left(i-1\right)$, and the current key request $K_{Supplement}\left(i\right)$ needs to queue up to wait for the time slot to be free. If $T_{get}\leq T_{arr}$, it means that the current key supplement request $K_{Supplement}\left(i\right)$ gets a response directly without queuing.

Then the average delay $\bar{T_{sum}}$ between the arrival of the key requests and the completion of the request processing is:

(10) $$\bar{T_{sum}}=T_{sum}/L_{req}$$

## 4. Simulation and Analysis

**Figure 6:**
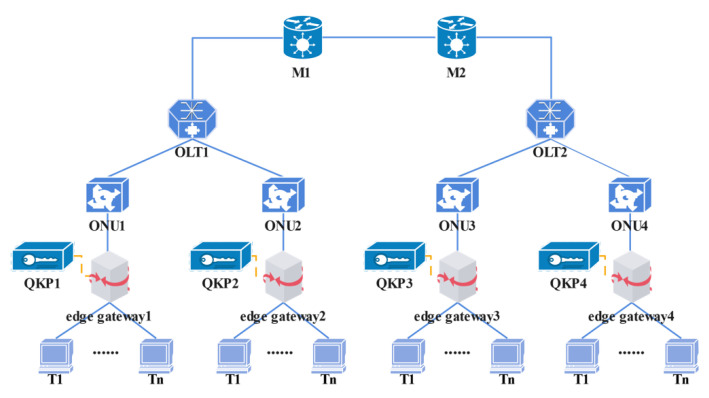
Simulated network topology.

In order to evaluate the performance of the DDKA-QKDN scheme for Q-IoT secured by QKDN, the simulation was performed based on the following settings. The simulation experiment is carried out on the network topology shown in Figure 6.

In the following performance evaluations, here are the common parameters. The arrival interval of the key service request meets the Poisson distribution and the Poisson distribution parameter of the request time interval is 5 s. The simulation scenario has four edge gateway nodes and the communication sender source edge gateway *ID* is generated randomly, so as the receiver destination edge gateway *ID*. The *ID* value is generated between 1 and 4, ID∈{1,2,3,4}. The quantum key quantity requirements $K_{qua}$ and quantum key security requirements $K_{sec}$ of the key service requests are also generated randomly. $K_{sec}\in \left\{1,2,3,4,5\right\}$ corresponds the lengths of quantum keys are 128 bits, 256 bits, 512 bits, 1024 bits, and 2048 bits. The benchmark capacity of the QKPs is 5120 bits and the initial remaining key amount $K_{sur}$ of the QKPs is pre-set $K_{sur}\in \left\{1280,2560\right\}\;\mathrm{bits}$. The edge gateway is connected to n IoT sensor devices. The trade-off degree ω of the quantity requirement and the security requirement of the key service is set to 0.5. The key generation rate $V_{gen}$ between OLT and ONU is 2560 bps, the link transmission rate $t_{tra}$ is 1280 bps. The factors affecting the performance of the proposed scheme are analyzed through simulations built in Matlab R2020b (The MathWorks, Natick, MA, USA). The simulation environment is 64-bit Windows 10. The hardware environment is Intel (R) Core (TM) i7-10700 CPU @ 2.90 GHz processor and 8.00 GB RAM.

This section evaluates scheme performance from two parameters, one is the average delay $\bar{T_{sum}}$ of the key service and another is the success rate SR of no-waiting requests. First, the average delay $\bar{T_{sum}}$ of key service refers to the average waiting time between the arrival of the key request and the completion of the request processing, which reflects the efficiency level of the key service of the scheme. Meanwhile, the success rate SR of no-waiting requests refers to the ratio of the key requests that can extract the keys from QKP upon arrival to the total key services, that is, the $T_{sum}$ of key requests is equal to the key transmission delay $T_{tra}$. The success rate of no-waiting requests SR reflects the ability of the QKP to directly provide the key request services. What is more, we measured the two parameters, $\bar{T_{sum}}$ and SR, according to the different key stream load, QKP thresholds, the trade-off degree ω of the quantity and security requirement of the key service.

### 4.1. Evaluation of DDKA-QKDN Scheme

**Figure 7:**
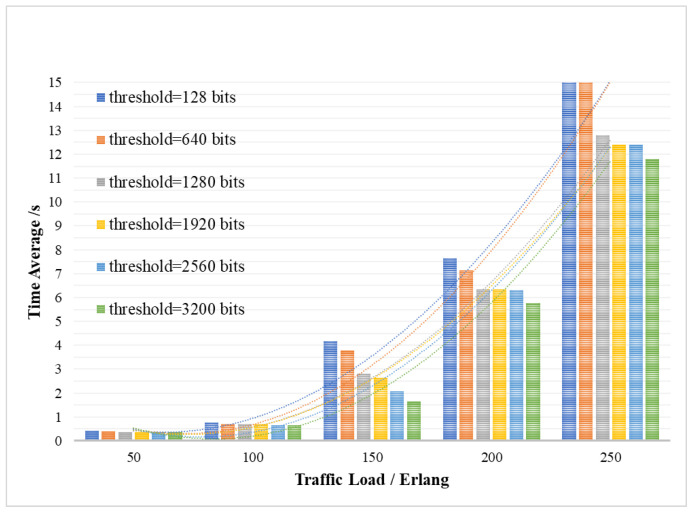
The average delay of the key services affected by traffic load and QKP threshold.

Figure 7 shows the efficiency level of the system, that is, the average key service delay $\bar{T_{sum}}$ of the entire system when the QKP differs in the minimum supplementary threshold $K_{threshold\_low}$ under different key service traffic loads. The average key service delay $\bar{T_{sum}}$ of the entire system averages the delay of all key requests for waiting $T_{sum}$, which consists of three parts: the queuing delay $t_{wait}$ of key requests waiting to obtain the key, the queuing delay $T_{wait}$ of QKP waiting for key supplement and the key transmission delay $T_{tra}$.The *Y*-axis of Figure 7 represents the average delay $\bar{T_{sum}}$ of key services, and the *X*-axis represents the traffic load of the key services.

As shown in Figure 7, the efficiency of key allocation of the whole system is related to the threshold $K_{threshold\_low}$ for the QKP key supplement and the key service traffic load $L_{req}$. As can be seen in Figure 7, as the key service traffic load $L_{req}$ gradually increases, the average delay $\bar{T_{sum}}$ of key service will gradually rise, and the efficiency of system key processing service will decline. This is because when the key resources are limited, it is necessary to wait for the dynamic supplement of the key resources. When continuous requests arrive, the time slice resource has not been able to be in an idle state, resulting in a corresponding increase in the delay of waiting for the supplement of key resources. It can also be seen that as the QKP low threshold $K_{threshold\_low}$ increases, the average key service delay $\bar{T_{sum}}$ and the efficiency of the system in processing key services will increase. The reason is that the key resources are gradually enriched and can provide more key request processing services, which improves the system efficiency.

**Figure 8:**
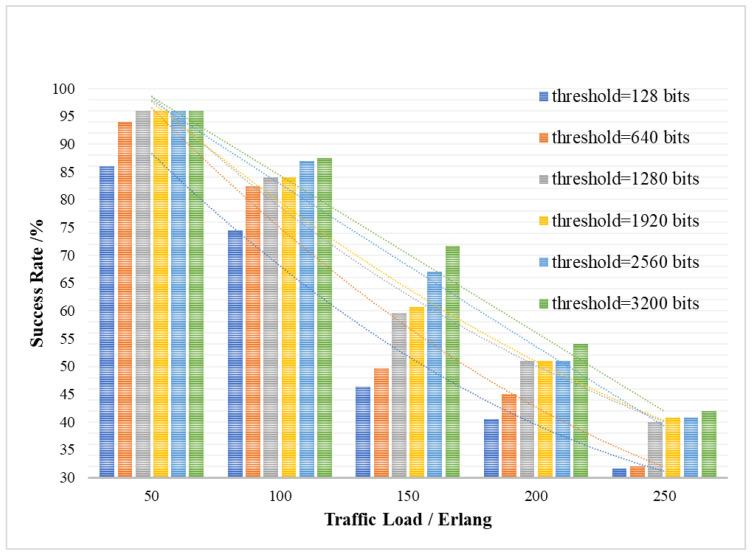
The success rate of the no-waiting requests affected by traffic load and QKP threshold.

Figure 8 shows the ability of QKP to directly provide the key request services, that is the success rate SR of no-waiting requests, under different key service traffic loads with variable minimum supplementary thresholds $K_{threshold\_low}$. The success rate SR of no-waiting requests is the ratio of key requests responded by QKP in time to all requests, which means that the queuing delay $t_{wait}$ of key requests waiting to obtain the key and the queuing delay $T_{wait}$ of QKP waiting for key supplement equals 0. The keys can be extracted from the QKP upon the arrival of the key request. The *Y*-axis in Figure 8 represents the success rate SR of no-waiting requests, and the *X*-axis represents the traffic load of the key services.

As shown in Figure 8, the ability of QKP to directly provide the key request services is related to the threshold $K_{threshold\_low}$ for QKP key supplement and the traffic load $L_{req}$ of the key services. It also can be seen that with the increase in traffic load $L_{req}$ of the key services, the success rate SR of no-waiting requests will decrease, which means the ability of QKP to directly provide the key request services will decrease. In the case of limited key resources, the increase in load will reduce the ability of QKP to directly provide the key request services. Meanwhile, as the QKP low threshold $K_{threshold\_low}$ increases, the success rate SR of no-waiting requests will increase, the reason of which is that the QKP key supplement process is added and the remaining key amount $K_{sur}$ of QKP is taken as a consideration. The dynamic key supplement can effectively enrich the key resources so that QKP can serve more quantum key requests.

**Figure 9:**
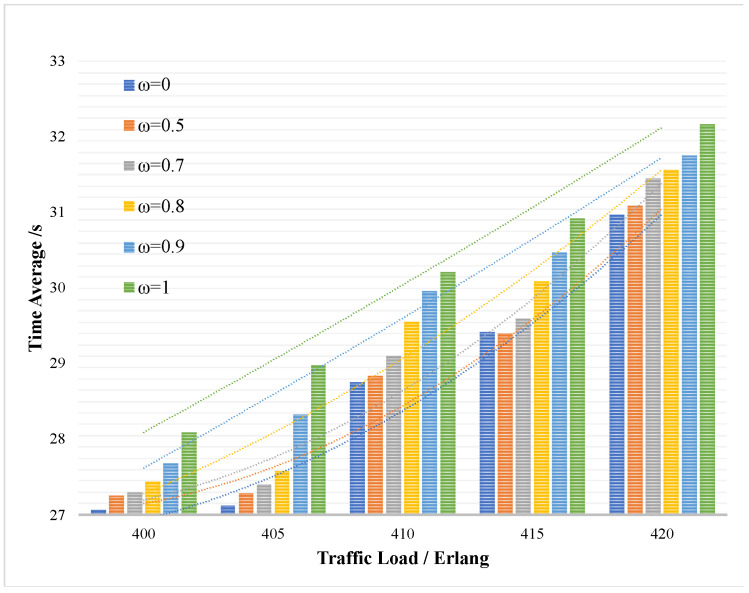
The average delay of the key services under different trade-off degrees ω.

In Figure 9, in order to verify the impact of the key request queuing strategy on the average delay $\bar{T_{sum}}$ of key service after the key request arrives, the same key supplement low threshold $K_{threshold\_low}$ is set and the trade-off degree ω in the sorting strategy is changed to adjust the proportion of the key demand $K_{qua}$ and the key security requirement $K_{sec}$ in the queuing standard. The trade-off degree ω of the quantity and security requirements in the sorting strategy is set to 0.5 in the initial state. In Figure 9, the trade-off degree ω is set from 0 to 1, ω∈[0,1], which means different requirements of the key quantity and the security of the system. When ω=0, it means that the sorting strategy only includes the key quantity requirement and when ω=1, it means that the sorting strategy only includes the security requirement. The *Y*-axis of Figure 9 represents the average delay $\bar{T_{sum}}$ of key service, and the *X*-axis represents the traffic load of the key services.

It can be seen that based on ω=0.5, the key requirement $K_{qua}$ and the key security requirement $K_{sec}$ account for the same proportion. When the proportion of key quantity requirement $K_{qua}$ decreases, $\bar{T_{sum}}$ will increase and the system efficiency will decrease. While the $\bar{T_{sum}}$ decreases and the system efficiency increases as the proportion of the key quantity requirement $K_{qua}$ increases, which situation is opposite to that before. However, at the same time, since the key security requirement $K_{sec}$ accounts for a lower proportion, the system security will decrease. Therefore, in practical application, the trade-off degree ω in the sorting strategy should be adjusted according to the application requirements.

### 4.2. Comparison of Different Schemes

The comparison schemes adopted in this section are the key allocation schemes proposed in [12,13,14]. In [12], the author proposed the RAKP scheme, which dynamically adjusts the key generation rate according to the key utilization of QKP. Ref. [13] proposed the KoD scheme, which uses the First-Fit algorithm to distribute keys through the control channel and data channel. Ref. [14] proposed a DSKP scheme, which selects the QKP with the lowest number of remaining keys to supplement first when the number of keys in QKP is less than a certain threshold.

In addition, the following three scenarios are considered. S1: when the key request arrives, the key application is not sorted according to the key requirement $K_{qua}$ and the key security requirement $K_{sec}$ weight, and at the same time there is no dynamic key supplement when the keys in QKP are insufficient; S2: for the key requests, they are not sorted according to the key requirement $K_{qua}$ and the key security requirement $K_{sec}$ weight; S3: when the keys in QKP are insufficient, there is no dynamic key supplement.

#### 4.2.1. Performance Comparison of Schemes under Different Traffic Loads

**Figure 10:**
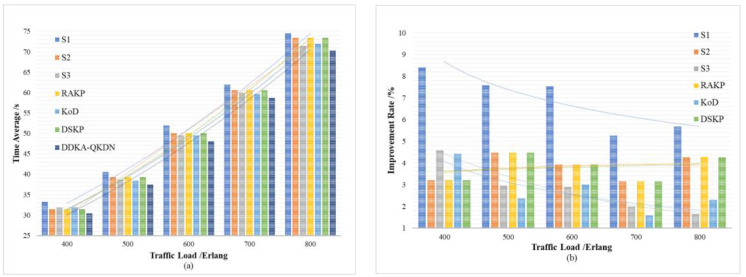
(**a**) The effect of the key service traffic load on average key service delay of each scheme; (**b**) The percentage improvement of the key service delay of the DDKA-QKDN scheme compared with other schemes.

**Figure 11:**
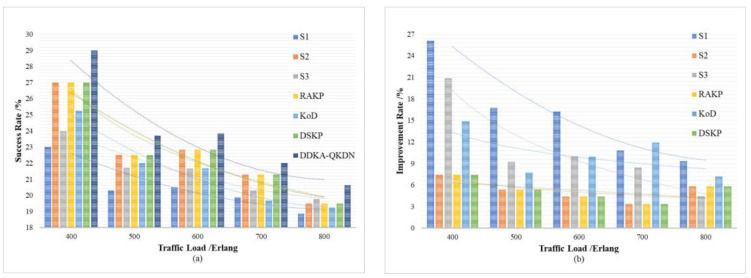
(**a**) The effect of the key service traffic load on the success rate of no-waiting requests of each scheme; (**b**) The percentage improvement of the DDKA-QKDN scheme in the success rate of no-waiting requests compared with other schemes.

In order to verify the improvement of the DDKA-QKDN scheme on the efficiency of key resource distribution, the same low threshold $K_{threshold\_low}$ of the QKP is set for key supplement and the trend of the average delay $\bar{T_{sum}}$ of key services of different schemes is compared with the change of the key service traffic load $L_{req}$. In order to verify the improvement of the DDKA-QKDN scheme on the ability of QKP to directly provide the key request services, we set the same low threshold $K_{threshold\_low}$ of the QKP for key supplement and compare the trend of the success rate SR of no-waiting requests of different schemes with the change of the key service traffic load $L_{req}$. In Figure 10b and Figure 11b, the percentage improvement refers to the ratio of the optimized value of the DDAK-QKDN scheme on this parameter to the value of the compared scheme.

As shown above in Figure 10 and Figure 11 comparing S1, S2, S3 and DDKA-QKDN schemes, it can be concluded that the current queuing strategy has little effect on the system efficiency and the improvement of the capability of QKP to provide key services, which is not greatly affected by the traffic load $L_{req}$. However, when the keys in QKP are insufficient, the key dynamic supplement improves the system efficiency and the ability of QKP to directly provide the key services, which decreases with the increase in $L_{req}$. An increase in $L_{req}$ means that $K_{qua}$ increases, and the possibility that the remaining key quantity $K_{sur}$ of QKP needs to be supplemented increases, so that more time slots are occupied. The idle time slots and the time slice resources are reduced so that the system efficiency is not significantly improved after $L_{req}$ is increased. However, the dynamic key supplement process takes the remaining key quantity $K_{sur}$ of QKP as a consideration, the dynamic supplement of key resources can significantly improve the capability of QKP to provide quantum key services.

Comparing this scheme with the RAKP scheme, KoD scheme, DSKP scheme, it can be seen that the DDKA-QKDN scheme improves the system efficiency by about 4% and the ability of the QKP to directly provide the key request services by about 7% compared with RAKP scheme and DSKP scheme. The RAKP scheme sorts the response order of key requests based on the key utilization of QKP, and the DSKP scheme sorts the response order of key requests based on the remaining key amount of QKP. In terms of improving the system efficiency, the sorting strategies of these two schemes are not as well as the DDKA-QKDN scheme. In addition, compared with the KoD scheme, the DDKA-QKDN scheme improves the system efficiency by about 2–5% and the ability of the QKP to directly provide the key request services by about 9–15%. The KoD scheme uses the First-Fit algorithm for key allocation, which is equivalent to the case of ω = 0 in the DDKA-QKDN scheme. According to the simulation results in the previous section, this strategy will have a certain effect on improving the system efficiency. However, the KoD scheme does not consider the key supplement process of QKP, and the excessive consumption of key resources after $L_{req}$ increases is not considered, which will lead to the decline of the ability of the QKP to provide the key request services. Meanwhile, in terms of improving the overall system efficiency, the effect is not equivalent to the DDKA-QKDN scheme and because the remaining key quantity $K_{sur}$ of QKP will drastically decrease as $L_{req}$ increases, and the time slice resources occupied by key supplement increase, it will result in less significant improvement in system efficiency after $L_{req}$ increases.

To sum up, the dynamic supplementary processing of QKP in the DDKA-QKDN scheme improves the overall efficiency of the system. The timely enrichment of key resources enables QKP to provide more key request processing services with the same time slice resources, which improves the system efficiency. Compared with the RAKP scheme and DSKP scheme, the queuing response strategy of the DDKA-QKDN scheme for requests improves the system efficiency more significantly. A key request with a large key demand $K_{qua}$ takes up more time to slice resources and link resources during processing, which will cause congestion in request processing. Therefore, when the request arrival time is the same, considering the key request with less key quantity requirement $K_{qua}$ first, which means key requests occupying with less time slice will improve the overall efficiency of the system.

#### 4.2.2. Performance Comparison of Schemes under Different QKP Thresholds

**Figure 12:**
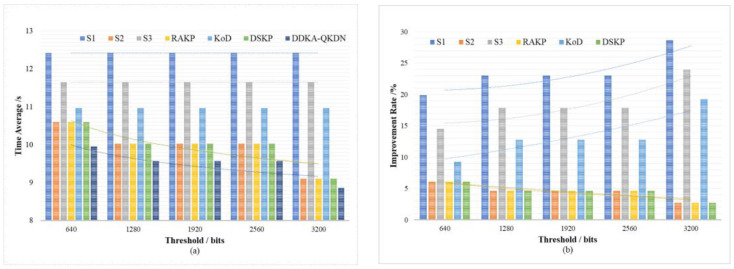
(**a**) The effect of the QKP key supplement threshold on average key service delay of each scheme; (**b**) The percentage improvement of the DDKA-QKDN scheme in the key service delay compared with other schemes.

**Figure 13:**
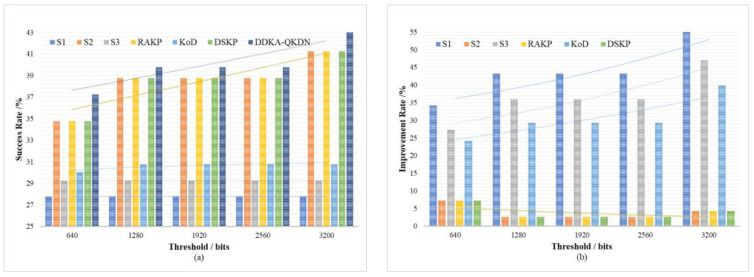
(**a**) The effect of the QKP key supplement threshold on the success rate of no-waiting requests of each scheme; (**b**) The percentage improvement of the DDKA-QKDN scheme in the success rate of no-waiting requests compared with other schemes.

For the sake of verifying the improvement of the DDKA-QKDN scheme on the efficiency of key resource allocation, we set the same traffic load $L_{req}$ of key services and compare the trend of the average delay $\bar{T_{sum}}$ of key services of different schemes with the change of the low threshold $K_{threshold\_low}$ for QKP key supplement. In order to verify the improvement of the DDKA-QKDN scheme on the ability of QKP to directly provide the key services, we set the same traffic load $L_{req}$ of key services and compare the trend of the success rate SR of no-waiting requests of different schemes changing with the low threshold $K_{threshold\_low}$ of QKP key supplement. In Figure 12b and Figure 13b, the percentage improvement refers to the ratio of the optimized value of the DDAK-QKDN scheme on this parameter to the value of the compared scheme.

As shown above in Figure 12 and Figure 13 comparing S1, S2, S3 and DDKA-QKDN schemes, we can come to the conclusion that after the requests are sorted according to the key requirement $K_{qua}$ and the key security $K_{sec}$ weight, the QKP threshold has little effect on the improvement of the system efficiency and the ability of QKP to provide the key services. The QKP threshold $K_{threshold\_low}$ mainly affects the dynamic supplement part of the key allocation. With the increase in the QKP threshold $K_{threshold\_low}$, the key resources become more abundant, which reduces the queuing delay of key requests and improves the system efficiency. When the keys in the QKP are insufficient, the dynamic key supplement improves the ability of QKP to directly provide the key services. Compared with the sorting strategy, the dynamic key supplement process will consider the remaining key quantity $K_{sur}$ of QKP, and the dynamic supplement of key resources can significantly improve the ability of QKP to provide the quantum key services.

Comparing this scheme with the RAKP scheme, KoD scheme, and DSKP scheme, it can be seen that the DDKA-QKDN scheme improves the system efficiency by about 5% and the ability of the QKP to directly provide the key request services by about 5% compared with the RAKP scheme and DSKP scheme respectively. In addition, compared with the KoD scheme, the DDKA-QKDN scheme improves the system efficiency by about 10–23% and improves the ability of the QKP to directly provide the key request services by about 23–37%. When the KoD scheme only adopts the First-Fit algorithm, it does not take the key supplement process of QKP, resulting in the change of the QKP threshold $K_{threshold\_low}$ in this scheme that has no effect on the improvement of the system efficiency. The response request sorting strategy adopted by the KoD scheme plays a limited role in the ability of the QKP to provide the key request services. However, the key supplement process of QKP in the DDKA-QKDN scheme can significantly improve the ability to provide quantum key services.

To sum up, the change of the QKP threshold $K_{threshold\_low}$ in the DDKA-QKDN scheme has a significant impact on the process of QKP key dynamic supplementation. Compared with the RAKP scheme and DSKP scheme, the queuing response strategy of the DDAK-QKDN scheme for requests has more stable variation during the change of threshold. Since the increase in the QKP threshold $K_{threshold\_low}$ mainly promotes timely enrichment of key resources, also because a key request with a large key quantity requirement $K_{qua}$ occupies more time slice resources and key resources, the abundance of key resources can reduce the delay of large demand requests, which can not only reduce the possibility of request congestion but also provide more key request processing services, so as to improve the system efficiency.

This section evaluates the performance of this scheme from multiple aspects and compares it with the RAKP scheme, KoD scheme, and DSKP scheme comprehensively. The simulation results show that the queuing response strategy of the DDKA-QKDN scheme has a significant effect on improving the efficiency of the system compared with the RAKP scheme and DSKP scheme. The DDKA-QKDN scheme puts great emphasis on the characteristics of requests and sorts them by the key quantity requirement $K_{qua}$ and key security requirement $K_{sec}$ so that the key resources can be allocated more efficiently. Compared with the KoD scheme, the dynamic key supplement process of the QKP in this scheme makes the time slice resources effectively utilized and improves the ability of the QKP to provide the key services, which leads to the improvement of the overall performance to a certain extent.

## 5. Conclusions

In IoT, to solve the problems of low key generation rate, the high deployment cost of QKD devices and the low storage capacity of IoT devices, the QKD network always needs to store quantum keys in the QKPs at the edge gateway. Due to the lightweight and efficient requirements of the IoT, it is urgent to improve the efficiency of the quantum key allocation. We creatively propose a dynamic on-demand key allocation scheme named DDKA-QKDN for Q-IoT, which fully considers the two processes of quantum key allocation and quantum key supplement. The scheme sorts the response sequence of the key requests based on the quantity and security requirements. Additionally, the scheme dynamically supplements the QKPs on-demand in consideration of the time slice resource, the remaining key amount of the QKPs and the key supplement request. The simulation results show that, compared with the RAKP and DSKP scheme, the DDKA-QKDN scheme can improve the system efficiency by up to about 5% and the ability of the QKP to directly carry the key request services by up to about 7%. In addition, compared with the KoD scheme, the DDAK-QKDN scheme can improve the system efficiency by up to about 10–23% and the ability of the QKP to directly handle the key request services by up to about 23–37%. Therefore, the scheme has higher key processing efficiency and can realize the balance between the QKD network quantum key resources and the security requirements of IoT terminal applications. In future work, the feedback obtained from each key allocation effect among multi-agents will be considered to dynamically adjust the key allocation scheme. Moreover, we will use the PADRES [25], a tool for privacy, data regulation, and security, to further test the security, privacy, and trust in the data that is processed.

## Figures and Tables

**Table 1:** Related works comparison.

References	Allocation Schemes			
	Application Scenarios of QKD	Major Consideration	Allocate on Demand	Key Supplement
Niu et al. [10]	classical QKD network	key size-driven wavelength	×	×
Wang et al. [11]	multiple domains	key allocation within arbitrary domains	×	×
Meng et al. [12]	Internet of Things	quantum key generation rate	×	×
Cao et al. [13]	software-defined optical network	key-updating based on time and data complexity	×	×
Wang et al. [14]	passive optical network	the number of keys in QKP	√	√
Cao et al. [15]	multi-tenant QKD network	secret-key-rate	√	×
Cao et al. [16]	multi-tenant QKD network	success probability of multi-tenant provisioning	√	×
Cao et al. [17]	QKD as a service (QaaS)	secret-key-rate	√	×
Zuo et al. [18]	classical QKD network	current resource usage of the network	√	√
Our scheme	Internet of Things	quantum key quantity and security requirements	√	√

**Table 2:** Abbreviation List.

Abbreviation	Descriptions
Qtra	Quantum transmitter
Qrec	Quantum receiver
QCh	Quantum Channel
PCh	Public Channel
VKP	Virtual Key Pool
T_i_	Internet of Things Terminal
G_i_	Edge Gateway
K_i_	Quantum Key
M_i_	Metropolitan Area Node
OTP	One Time Password

**Table 3:** Mathematical symbol list.

Mathematical Symbol	Descriptions
Sec	security level
$est\left(K_{i}\right)$	response weight value
ω	trade-off degree of the quantity and security requirements
$K_{qua}$	quantum key quantity requirement
$K_{sec}$	quantum key security requirement
$K_{sur}$	remaining key amount of the QKP
$K_{threshold\_low}$	the low threshold of the QKP
$K_{threshold\_high}$	the high threshold of the QKP
$t_{arr}$	arrival time of the requests
$L_{req}$	key stream load
$T_{tra}$	the key transmission delay
$V_{gen}$	key generation rate
$T_{sum}$	the delay of the key request for waiting
$\bar{T_{sum}}$	the average delay of each key request for waiting
$t_{wait}$	the queuing delay of key requests waiting to obtain the key
$t_{slot}$	time slot
λ	the arrival frequency of the key requests
$T_{wait}$	the queuing delay of QKP waiting for key supplement
$t_{get}$	the time when the last key request obtained the keys
$t_{tra}$	the link transmission rate
$T_{get}$	the time when the last QKP key supplement request obtained the key
$T_{arr}$	the arrival time of the current QKP key supplement request
SR	the success rate of no-waiting requests

**Table 4:** The pseudo code of the DDKA-QKDN scheme.

**Scheme:** DDKA-QKDN: Dynamic on-Demand Key Allocation Scheme	
**Input**: key request r (source gateway s, destination gateway d, key quantity requirement $K_{qua}$, key security requirement $K_{sec}$, arrival time $t_{arr}$), key stream load $L_{req}$, QKP P (key surplus $K_{sur}$, high threshold $K_{threshold\_high}$, low threshold $K_{threshold\_low}$), transmitting delay $T_{tra}$, key generation rate $V_{gen}$.	
**Output**: the average delay of each key request for waiting $\bar{T_{sum}}$.	
1	**for** all the key request $r_{i}$ **do**
2	calculate the weight value of the key quantity requirement $K_{qua}$ and the security $K_{sec}$ requirement $est\left(K_{i}\right)$;
3	**end for**
4	**for** all the key request $r_{i}$ **do**
5	sort in ascending order of the arrival time $t_{arr}$ and the weight value $est\left(K_{i}\right)$;
6	**end for**
7	**for** each edge gateway $x_{i}$ **do**
8	consider the corresponding QKP $P_{ij}$, according to the destination gateway $x_{j}$ of each key request $r_{i}$;
9	**if** key quantity $K_{qua}\;$ > key surplus $K_{sur}$ of the QKP $P_{ij}$ **, **then****
10	record this key request $r_{i}$ requiring key supplement for the QKP as $flag_{i}$;
11	record the waiting time $t_{wait}$ and update the time $t_{arr}$ till applying for the key supplement;
12	**else** record the waiting time $t_{wait}$ and the time to obtain the key $t_{get}$;
13	update key surplus $K_{sur}$ of the QKP $P_{ij}$;
14	**end if**
15	**if** key surplus $K_{sur}$ ≤ $K_{threshold\_low}$, **then**
16	supplement keys for the QKP $P_{ij}$ in the interval time between two key requests, update key surplus $k_{sur}$ of the QKP $P_{ij}$;
17	stop supplement keys till key surplus reach $K_{threshold\_high}$;
18	**end if**
19	**if** key supplement $flag_{i}$ under the same $OLT_{i}$, **then**
20	sort in ascending order of the arrival time $t_{arr}$ and the weight value $est\left(K_{i}\right)$;
21	record the waiting time $T_{wait}$;
22	**end if**
23	**end for**
24	**for** all the key request $r_{i}$ **do**
25	Calculate the average time for waiting $\bar{T_{sum}}=\left(t_{wait}+T_{wait}+T_{tra}\right)/L_{req}$;
26	**end for**
